# Impact of a Commercially Available Low Carbon Renewable Diesel Fuel on the Light-Off and Light-Down Characteristics of a Diesel Oxidation Catalyst

**DOI:** 10.1021/acsomega.2c03744

**Published:** 2022-08-23

**Authors:** Jordan E. Easter, Martin L. Wissink, Vicente Boronat Colomer

**Affiliations:** Energy Science and Technology Directorate, Oak Ridge National Laboratory, P.O. Box 2008, Oak Ridge, Tennessee 37831, United States

## Abstract

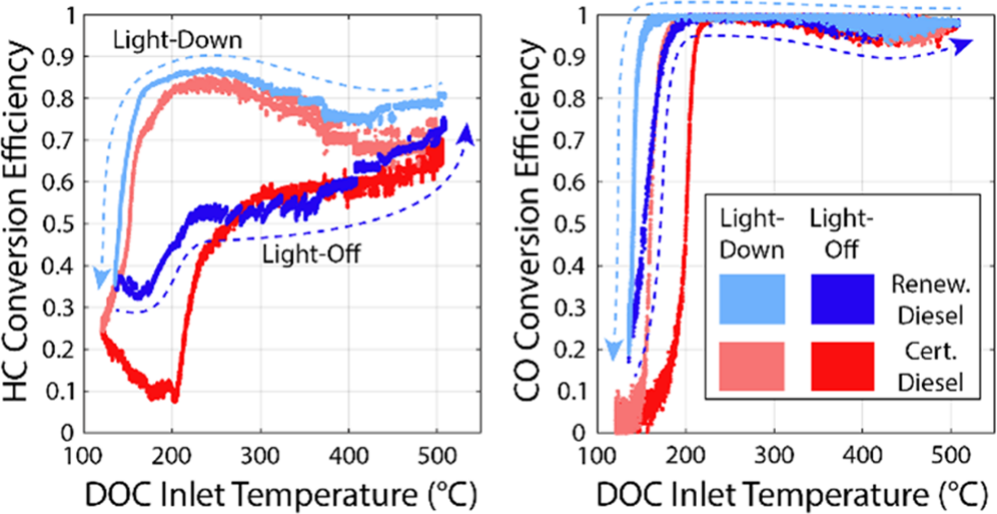

Meeting future greenhouse gas emissions targets in transportation may require transition in part to renewable low carbon fuels to power the medium- and heavy-duty sectors. At this moment, market renewable low carbon diesel fuels are available and integrated with the fueling infrastructure in select areas. Though this is encouraging, little is known about the impact these renewable diesel fuels may have on modern aftertreatment systems and their ability to convert toxic emissions. This work explores the impact of a renewable hydrotreated vegetable oil (HVO) diesel fuel on catalyst light-off and light-down of a diesel oxidation catalyst (DOC) with a production diesel engine over ramp rates reflective of real-world operation. Hydrocarbon (HC) and carbon monoxide (CO) emissions were investigated using various exhaust analyzing instruments placed before and after the model γ-Al2O3 DOC: Flame ionization detector (FID), nondispersive infrared (NDIR), and Fourier transform infrared spectroscopy (FTIR). The results of this work conclude that HC and CO conversion during catalyst light-off and light-down is significantly impacted by the fuel properties unique to the mostly paraffinic renewable HVO diesel, with light-off and light-down of the catalyst being improved for the renewable diesel fuel with respect to a certification diesel fuel for all ramp rates explored. Compared to certification diesel, HVO diesel reduced steady-state DOC-out HC and CO at idle by >50% and reduced the 50% conversion temperature (T50) during light-off by 45 °C for both HC and CO at a 20 °C/min ramp rate.

## Introduction

Transportation is the single largest contributor to anthropogenic greenhouse gas emissions within the United States, comprising 28% of estimated emissions. Medium- and heavy-duty (MD/HD) trucking contributes to a significant 23% of the greenhouse gas emissions in this sector.^1^ Though electrification is promising in its ability to address greenhouse gas emissions for both medium- and heavy-duty trucks, given current issues such as the lack of a charging infrastructure and high battery cost,^2^ there are still concerns regarding how quickly this sector will electrify. One near-term and low-cost solution for reducing greenhouse gas emissions in MD/HD trucking is to transition conventional petroleum-derived diesel fuel to a fully renewable low carbon diesel fuel such as a retail available renewable hydrotreated vegetable oil (HVO). When considering a full life cycle assessment, including feedstock production, transportation to refineries, refinement, and combustion efficiency of the engine burning the fuel, significant reductions in greenhouse gas emissions are observed for renewable HVO diesel fuels. With detailed reports in the literature finding improvements in greenhouse gas emissions ranging from 30 to 80% for HVO renewable diesel with respect to conventional fossil-derived diesel fuel.^3−5^

HVO renewable diesel fuel is often produced through the refinement of heavy chain hydrocarbons from waste vegetable oils and animal fats into lighter alkanes. They are mostly paraffinic and devoid of sulfur, oxygen, aromatics, and ash-forming materials. Many of the properties of renewable HVO diesel fuels allow them to integrate easily into the current fuel infrastructure and work well in modern diesel engines without blending requirements.

In addition to addressing greenhouse gas emissions, reduction of other regulated harmful criteria pollutants such as unburned hydrocarbons (HCs), nitrogen oxides (NO*_x_*), and carbon monoxide (CO) must be maintained with the use of the HVO renewable diesel fuel if it is to function as a true drop-in replacement. Diesel engines today utilize a complex aftertreatment system with various catalysts and sophisticated controls to meet strict regulatory standards for those harmful criteria pollutants just mentioned. Though research has been conducted to evaluate the impact of HVO renewable diesel fuel on engine-out emissions and combustion efficiency,^6−10^ little is reported on the impact this fuel may have on aftertreatment performance.^11−13^

Specifically, the diesel oxidation catalyst (DOC) used to oxidize CO and HC may be impacted by the shift to a renewable HVO diesel. Given the low exhaust temperatures of diesel engines and the wide-ranging duty cycle in MD/HD applications, light-down of the DOC may occur during normal operation. This can be a large source of tailpipe HC and CO emissions and an area where fuel impact on the DOC is of much importance. It is well known that fuel chemistry plays a significant role in affecting the light-off characteristics of a DOC. This dynamic is complex and well researched using highly controlled bench reactor-type setups in the literature. The individual HC family and carbon number, a mixture of HC, and amount of HC present, among other factors, have been found to impact DOC light-off and overall HC/CO conversion efficiency.^14−19^ The HVO renewable diesel is expected to vary in the amount of total HC engine-out, with past research suggesting that the use of HVO renewable diesel may result in a moderate reduction in emissions as a result of improved fuel reactivity (cetane number).^6,13,20^ It is also expected to vary in hydrocarbon composition in a manner that may impact DOC activity.^21^ For example, HVO renewable diesel completely lacks an aromatic component, a HC family known to inhibit oxidation of other HC given the strong interaction of aromatics with the catalyst surface, thereby out competing other fuel species for adsorption on active catalyst sites.^22−24^

Another aspect of light-off and light-down of a DOC is the rate at which the temperature ramp occurs. Most of the present literature has evaluated catalyst performance over long ramp times where the system may reach thermal and chemical equilibrium at each operating temperature. This allows for a deeper understanding the fundamental kinetics of the system. However, this does not capture the transient behavior and catalyst dynamics seen in real-world systems where the DOC inlet temperature changes quickly. Some research works have explored faster transients using modern production engines, with unique results relative to those observed on bench reactors with long ramp rates.^25,26^ These works observed large shifts in catalyst light-off temperature, significant differences between catalyst light-off and light-down performance, and potential HC storage followed by a subsequent release at higher temperatures. Though these works have contributed to an improved understanding regarding DOC performance under real-world-type conditions, they are limited in number and none explore renewable HVO diesel fuels.

In this work, a conventional diesel engine and several transient ramp rates are used to explore the impact HVO renewable diesel has on engine-out HC/CO emissions and catalyst light-off and light-down performance. This information is currently missing from the literature and is useful in determining if the commercially available HVO renewable diesel may address both greenhouse gas and other criteria pollutants, such as unburned HC and CO, without changes to the current engine and aftertreatment designs.

## Materials and Methods

The engine setup used for this study included a 2007 four-cylinder General Motors 1.9 direct-injection turbocharged diesel engine with specifications given in Table 1. The stock engine control unit was replaced with a flexible data acquisition and control system from the National Instruments Powertrain Control Group. This system allows for full control of all engine parameters, including the simulation of a pedal input. For more detail on this engine setup, refer to previous publications.^27,28^

**Table 1:** Engine Specifications^a^

number of cylinders	4
bore (mm)	82.0
stroke (mm)	90.4
connecting rod length (mm)	145.4
displacement (L)	1.91
compression ratio	16.5
intake valve opening [°CA aTDC]	344
intake valve closing [°CA aTDC]	–132
exhaust valve opening [°CA aTDC]	116
exhaust valve closing [°CA aTDC]	–340
rated power [kW]	110
rated torque [Nm]	315

a Acronyms: aTDC = after top dead center, CA = crank angle.

To understand the impact of the HVO renewable diesel on DOC performance and engine-out emissions, a 2007 certification diesel was used as a baseline fuel. Fuel properties of interest to this study are provided in Table 2. Additional relevant fuel properties, including the full distillation curve, are provided in Table S1 and Figure S1 in the supporting material.

**Table 2:** Fuel Properties^a^

		boiling range (°C)							
fuel	cetane number	T10	T50	T90	aromatic (mass %)	paraffin (mass %)	density (g/mL)	LHV (kJ/g)	H/C (mol/mol)
2007 cert. diesel	42.8	208	258	309	31.1	29.6	0.854	42.7	1.773
HVO renew. diesel	74.8	260	281	293	0.1	96.9	0.780	43.9	2.113

a Acronyms: T10 = temperature at 10% distillation, T50 = temperature at 50% distillation, T90 = temperature at 90% distillation, LHV = lower heating value.

As expected, the HVO renewable diesel is nearly devoid of aromatics. It is also interesting to note the tighter boiling range for the HVO renewable diesel with respect to the 2007 certification diesel. This would suggest differences in the hydrocarbon chain lengths making up the paraffinic components between the two fuels. Given that aromatic content and hydrocarbon chain length are known factors to influence DOC performance,^15,17,18^ it is expected that DOC performance would be influenced by the transition to the HVO renewable diesel from the 2007 certification diesel.

The HVO renewable diesel has a significantly higher cetane number with respect to the 2007 certification diesel. An increase in the cetane number indicates an increase in fuel reactivity, and HC and CO emissions are expected to decrease as the cetane number increases as a result of more complete oxidation of fuel species and a reduction in the ignition delay.^29−32^

The model DOC used in this work was a 300 cells/in^2^ cordierite coated with 100 g/ft^3^ Pt on 160 m^2^/g γ-Al_2_O_3_. This model DOC has been used in previous research works.^33^ The DOC was instrumented with thermocouples at the inlet and outlet and four axial locations within the catalyst from the inlet to the outlet. A diagram of this instrumentation is provided in the supplemental information, Figure S2.

DOC light-off and light-down were explored through exhaust temperature ramp cycles using a simulated pedal input, examples of which are given in the supporting information, Figure S3. This testing included cycling from a high load of 17 bar brake mean effective pressure (BMEP) to a low load of 0 bar BMEP and back again to 17 bar BMEP using a specified ramp rate. A 10 min hold time was used when either the high-load or low-load operating point was reached.

In this work, two ramp rates were used. The slower ramp rate of 20 °C/min was selected as it represents a reasonable increase in the ramp rate relative to conventional reactor bench DOC testing^34^ while maintaining spatial homogeneity of temperature within the catalyst prior to light-off. The faster ramp rate of 400 °C/min was selected for use in this study as it represents the fastest ramp rate by which transient measurement fidelity of the emission analyzers could be maintained.

Each combination of fuel and the ramp rate was evaluated three times to ensure repeatability. The recorded results were very consistent between tests and only one of the three tests is shown for each case to improve the clarity of the figures.

In addition to transient ramps, steady-state emissions were taken at the 0, 2.5, 6.5, and 17 bar BMEP load conditions. Measurements were taken after the engine had operated for 10 min at the desired operating condition. Hysteresis was investigated by performing the steady-state measurements first in descending order for DOC light-down and then in ascending order for DOC light-off.

Fourier transform infrared (FTIR) spectroscopy was used to speciate various exhaust constituents at both the engine-out and post DOC location. This was completed using an MKS MultiGas Model 2030 HS Analyzer. Total unburned HC emissions at both the engine-out and post DOC location were measured with a California Analytical Instruments (CAI) heated flame ionization detector (FID). Though CO emissions presented in this paper were taken through FTIR spectroscopy, these numbers were confirmed for the engine-out location through a CAI nondispersive infrared (NDIR) instrument. Exhaust samples were passed through heated filters and carried to each instrument through heated lines maintained at 191 °C. Where shown, error bars indicate the highest and lowest measured values of all runs for a given experimental task.

**Figure 1:**
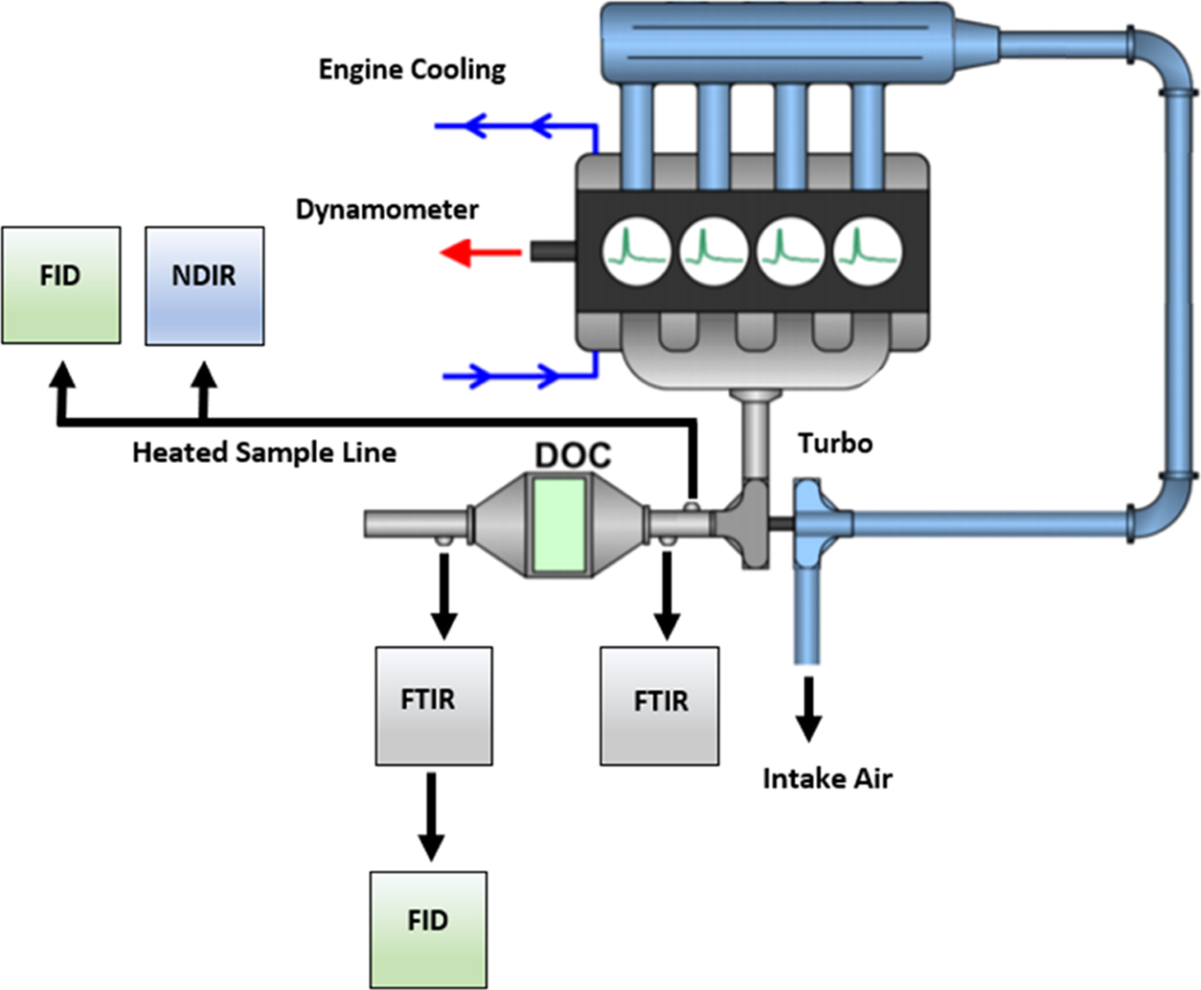
Schematic of the experimental setup. Adapted with permission from Prikhodko et al.^28^

A schematic of the experimental setup, including the location of emissions measurements, is provided in Figure 1. This schematic is adapted from a previous publication.^28^ Exhaust emissions were sampled at the engine-out and post DOC locations. The FID measurements taken after the DOC were uniquely sampled through the exhaust of the FTIR sampling at the post DOC location given test cell constraints.

## Results and Discussion

### Steady-State Emissions

**Figure 2:**
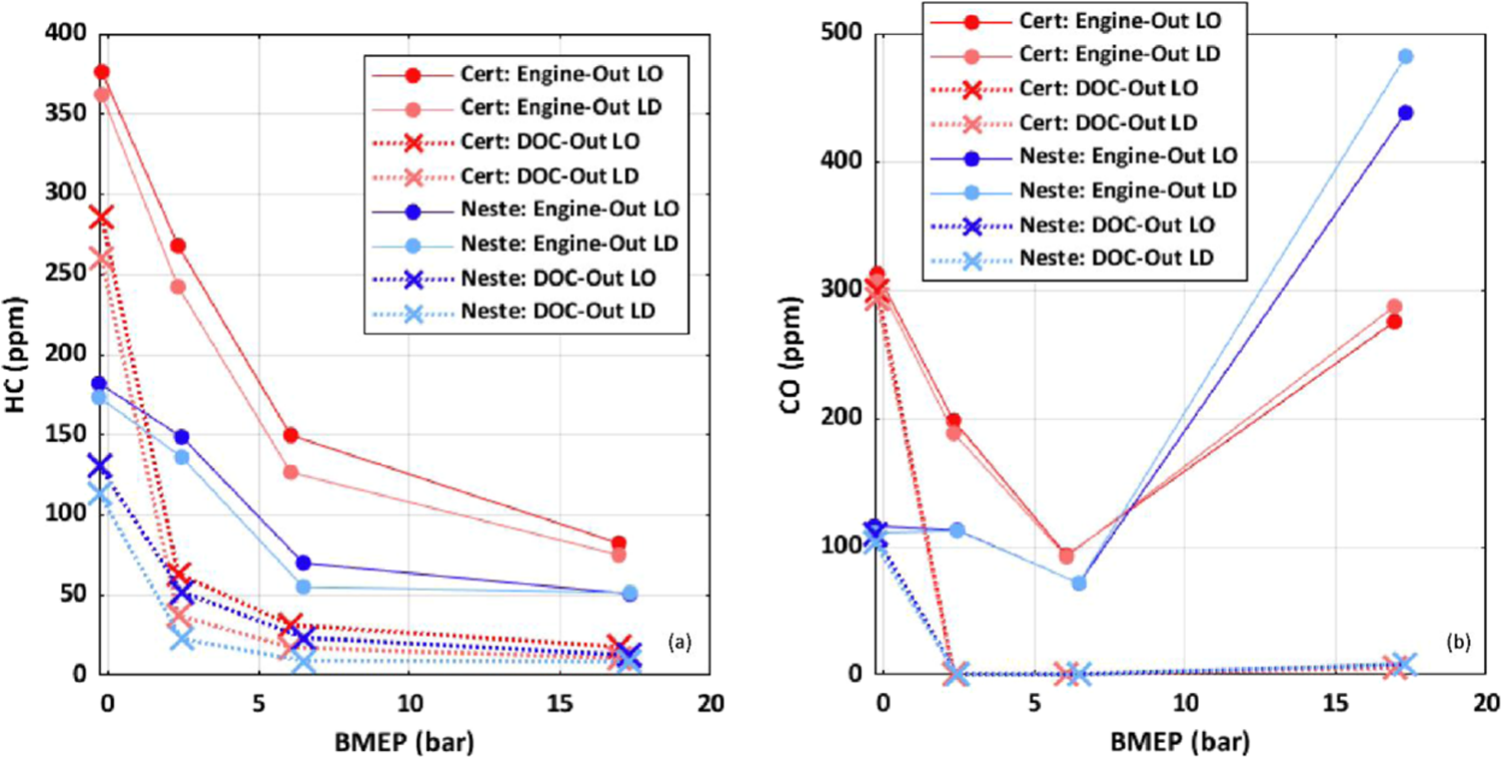
Engine-out and DOC-out HC and CO emissions at steady-state operation. LO = light-off, LD = light-down.

HC and CO engine-out and DOC-out emissions at all steady-state conditions evaluated are presented in Figure 2. For both fuels, HC engine-out emissions increased as load decreased, whereas CO decreased with load down to ∼7 bar BMEP and then increased again as load continued to drop. These trends are likely explained by shifts in the equivalence ratio as the load is changed, as will be discussed later.

For nearly all load conditions, HC and CO emissions were lower for the HVO renewable diesel than for the 2007 certification diesel. Previous works have also reported reduced HC and CO engine-out emissions when using HVO renewable diesel.^6,20^ This may be explained by the improved fuel reactivity, as noted by the higher cetane number, for the HVO renewable diesel when compared to the 2007 certification diesel. The improved fuel reactivity should reduce ignition delay, resulting in less fuel burned in the pre-mixed phase, known for higher HC and CO emissions with respect to the diffusion phase.^29,30^ Additionally, the increase in fuel reactivity should result in improved rates of CO and HC oxidation, preventing over-lean or low-temperature combustion zones where HC and CO emissions are more likely to form.^31,32^ However, there is one condition where the reverse is true. Engine-out CO emissions were higher for the HVO renewable diesel at 17 bar BMEP. This may be explained by differences in the equivalence ratio between the two fuels resulting from fuel property differences, as will be discussed later. However, given the high conversion efficiency (98%), the post DOC emissions were very low for both fuels.

As expected, conversion over the DOC is temperature- and therefore load-dependent. DOC conversion increases as load increases with high CO and HC conversion at loads above the 0 bar BMEP low-temperature idle condition. This low-temperature condition at 0 bar BMEP highlights an important area where the fuel properties matter for steady-state operation. As a result of poor DOC conversion at this condition, tailpipe HC and CO emissions are much higher for the 2007 certification diesel with respect to the HVO renewable diesel given the differences in engine-out HC and CO emissions. There is a 64% reduction in tailpipe CO emissions and a 55% reduction in tailpipe HC emissions when using the HVO renewable diesel at the 0 bar BMEP operating condition.

### Engine-Out Emissions During 20 °C/min Ramp

**Figure 3:**
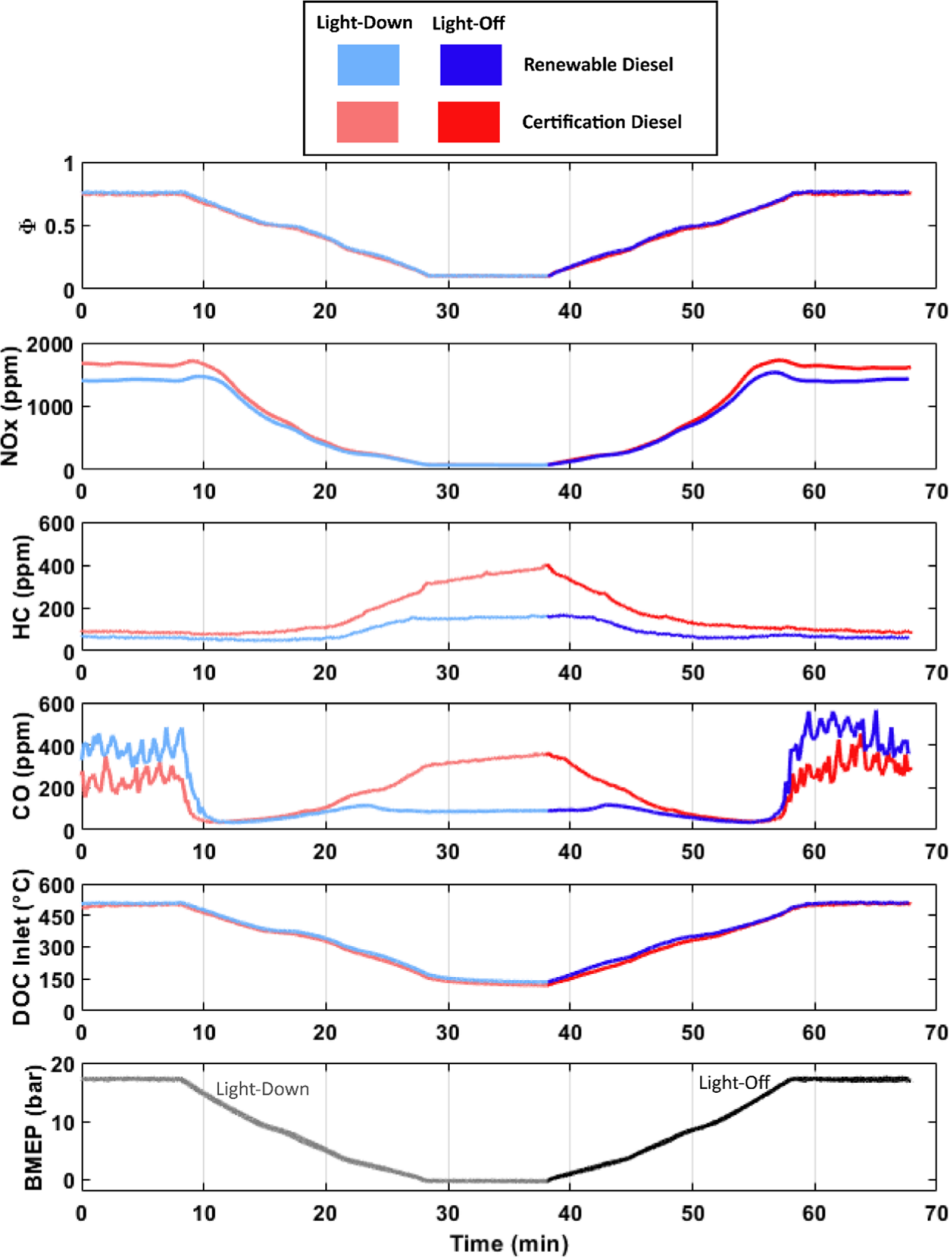
Engine-out emission comparison during the 20 °C/min ramp.

The engine-out emission constituents during the slower 20 °C/min ramp rate are given for both fuels in Figure 3. The HC and CO engine-out emissions are higher for the 2007 certification diesel with respect to the HVO renewable diesel for nearly the entire ramp cycle, with the only exception being the CO emissions at the highest load operating condition. Conversely, engine-out NO*_x_* was higher for certification diesel at high load but was similar between the two fuels at idle. Select species from the FTIR and a total hydrocarbon comparison between FTIR and FID are shown in the supporting information, Figure S4.

The improved fuel reactivity for the HVO renewable diesel should result in improved HC/CO emissions for reasons discussed previously. The high-load operating condition where this trend reverses for CO emissions may be explained by slight differences in engine operating parameters. The equivalence ratio, given by Φ, is slightly higher for the HVO renewable diesel with respect to the 2007 certification diesel over the entire ramp. This difference in Φ between the two fuels is likely a result of the engine control method used for this work. Because of differences in density and the heating value, the injection duration schedules were multiplied by a scalar factor to match the load between the two fuels. However, the fuels also vary slightly in the H/C ratio, which impacts the stoichiometric air/fuel ratio. It is possible in principle to modify the desired intake pressure tables to account for this, but that is unlikely to happen in a real-world scenario where the engine controller does not have access to detailed fuel properties, and we preferred to match the pumping loop boundary conditions rather than the equivalence ratio. As engine operation becomes richer, increased CO emissions become more likely as less oxygen is available for complete oxidation of HC species and the oxidation process is stopped at the formation of CO.

Considering again Figure 3, for both fuels, as the ramp moves through the light-down event, from a high to low-load operating condition, there is a shift to increased HC and CO emissions at lower catalyst temperatures and leaner conditions. It is expected that HC and CO emissions would generally increase as the load is reduced as leaner operation can result in potential over leaning and lower in-cylinder temperatures preventing complete oxidation of fuel species.^30,31,35^

### Impact of the Ramp Rate

**Figure 4:**
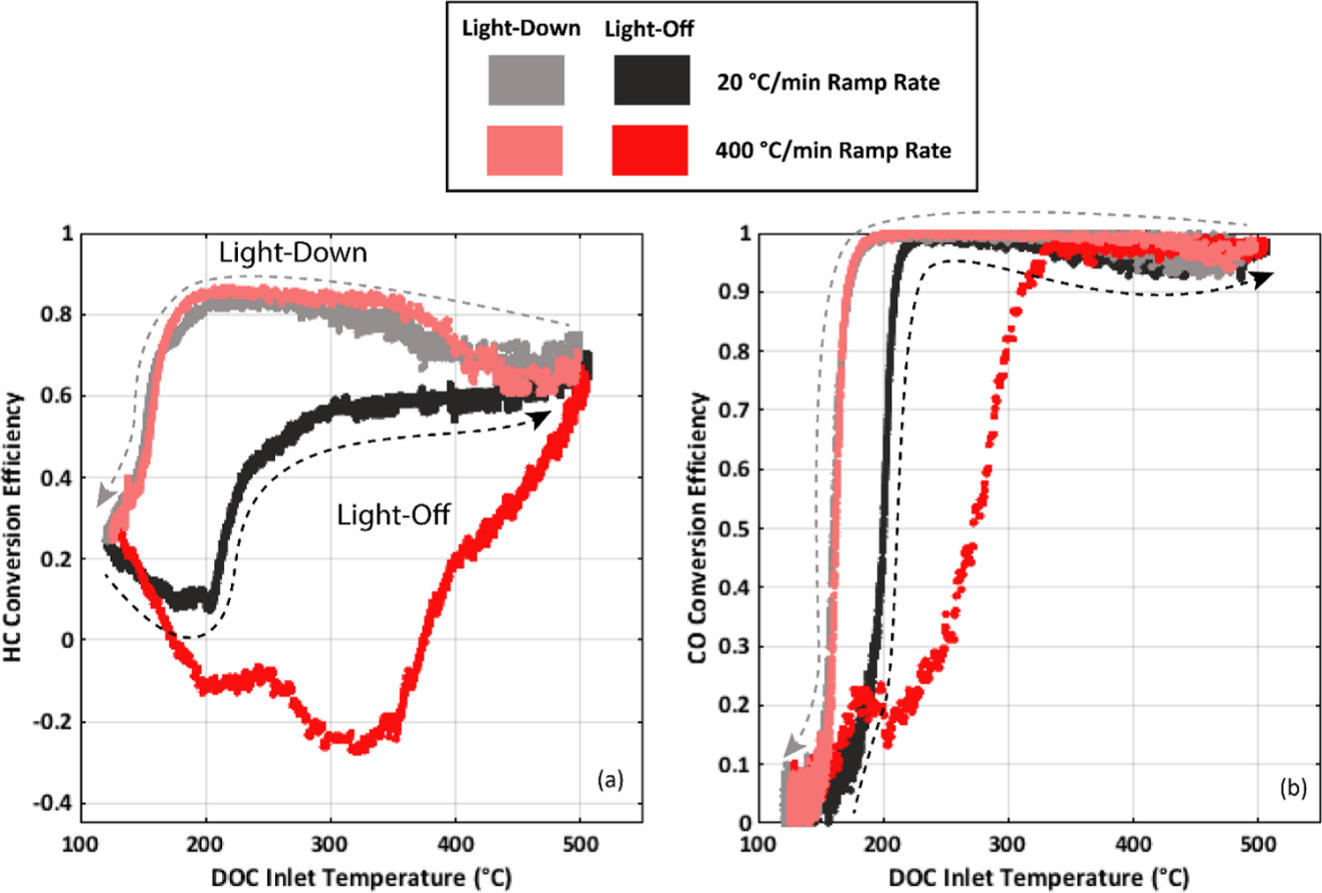
Comparison of HC (a) and CO (b) conversion efficiencies during light-off and light-down as a function of DOC inlet temperature during the two different ramp rates. A comparison is made with the 2007 certification diesel.

The impact of the ramp rate on DOC conversion efficiency for the 2007 certification diesel is reviewed in Figure 4. Regardless of the ramp rate, light-down for both HC and CO emissions occurs at a lower catalyst inlet temperature than light-off. This has been observed in other research works exploring hysteresis in light-off and light-down events for a DOC.^25,26^ It is expected that during light-down events, catalyst temperatures remain higher internally due to sustained exothermic reactions. This increase in internal catalyst temperatures would result in an improved CO and HC conversion efficiency. There are also some mechanistic effects likely at play. One explanation could be differences in adsorbed HC and CO species on the catalyst active sites during the ramp events. HC and CO chemisorption to active sites is highly dependent on temperature. At low temperatures, below catalyst light-off, HC and CO species are more likely to be adsorbed to precious metal surfaces blocking O_2_ adsorption.^14,36−39^ This would negatively impact HC and CO oxidation. It is possible that during light-off, it takes additional time for the relevant HC and CO to be removed from active sites, so conversion is impacted even beyond the light-off catalyst temperature. It is also possible that some of the less volatile HC species condense onto the DOC substrate at lower temperatures, which vaporize during initial heating and result in the negative conversion efficiencies seen during light-off. However, during light-down, conversion remains high even as temperatures are reduced. It seems plausible that during light-down fewer active sites for O_2_ adsorption are blocked by adsorbed HC and CO molecules as HC and CO species were likely removed by extended operation at higher catalyst temperatures.

The increase in the ramp rate from 20 to 400 °C/min resulted in an increased light-off temperature and decreased overall conversion performance for both CO and HC emissions. It is expected that with the increase in the ramp rate, catalyst substrate heating would be reduced given a reduction in the time for heat transfer to occur. Additionally, the faster ramp rate provides less time for adsorbed HC and CO species to be removed as temperatures increase.

**Figure 5:**
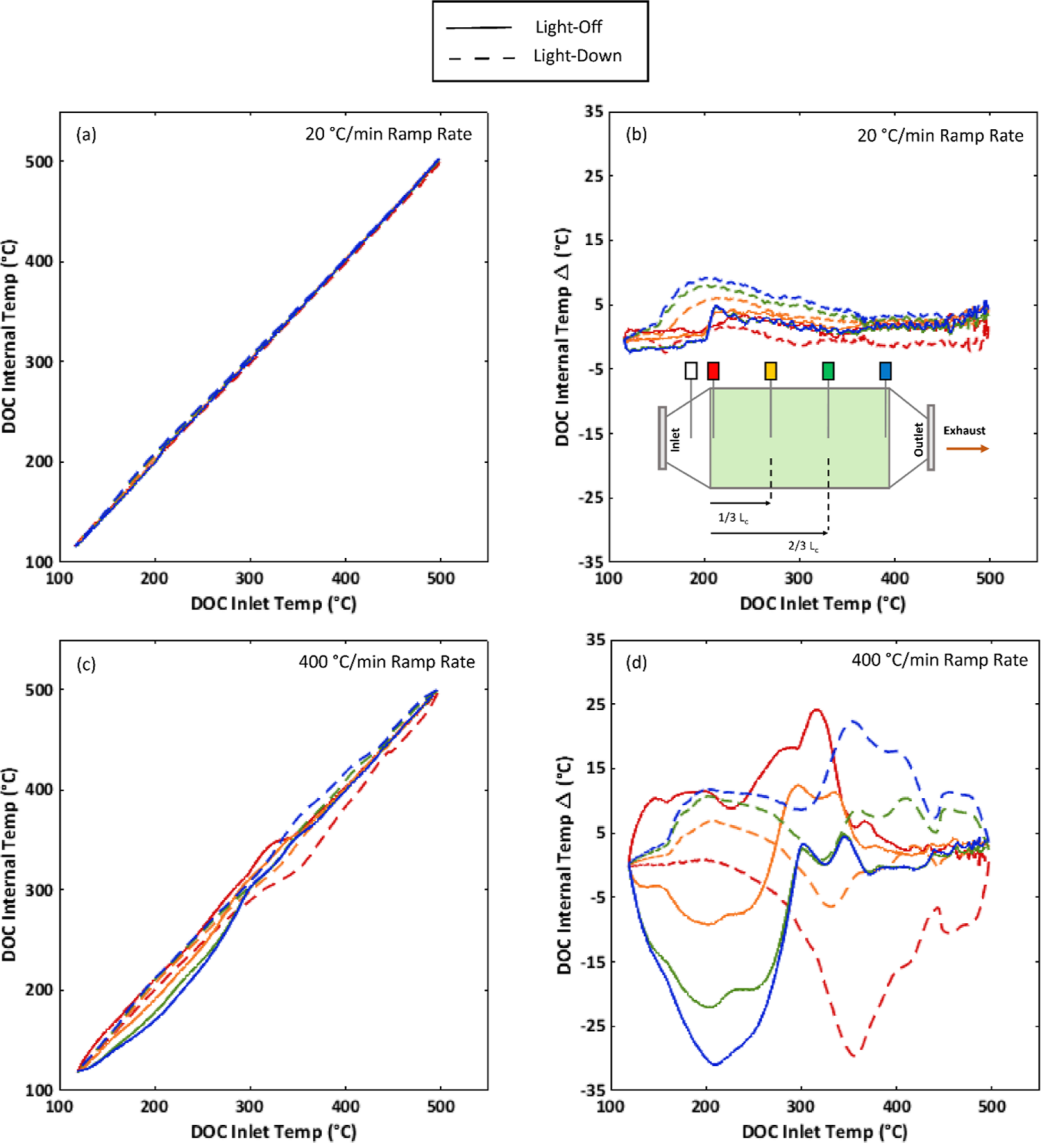
DOC temperatures during differing ramp rates using the 2007 certification diesel: (a) 20 °C/min ramp rate and DOC internal brick temperatures, (b) 20 °C/min ramp rate and DOC internal brick temperature difference from the DOC inlet temperature, (c) 400 °C/min ramp rate and DOC internal brick temperatures, and (d) 400 °C/min ramp rate and DOC internal brick temperature difference from the DOC inlet temperature.

Internal catalyst temperatures are plotted in Figure 5. The line colors used for all four plots correspond to the axial thermocouple location as color-coded on the DOC graphic in subplot (a). The DOC internal temperature Δ, as plotted in the graphs on the right-hand side of Figure 5, is calculated as the difference between the temperature within the catalyst brick and the inlet DOC temperature.

Internal temperatures are on average higher for the light-down event with respect to light-off during the slower 20 °C/min ramp rate. This suggests that sustained reactions are likely occurring within the catalyst during light-down, contributing to improved HC and CO conversion during light-down.

As the ramp rate increases to the 400 °C/min condition, temperatures within the DOC become more varied during both the light-down and light-off conditions. During light-off, there is mostly a reduction in catalyst internal temperatures with respect to the slower ramp rate. This may explain in part the reduced DOC performance during light-off as the ramp rate was increased. The lower internal catalyst temperatures for the faster ramp rate were expected given the reduced time for heat transfer to occur. There is an exception, around an inlet DOC temperature of 250 °C, internal catalyst temperatures spike beyond values recorded for the slower ramp rate. It may be that at this point HC and CO stored either on active sites or the substrate are exhausted, and a sudden transition to exothermic reaction occurs, resulting in a temperature spike that then settles as the DOC inlet temperature continues to climb.

### Impact of Fuel Type

**Figure 6:**
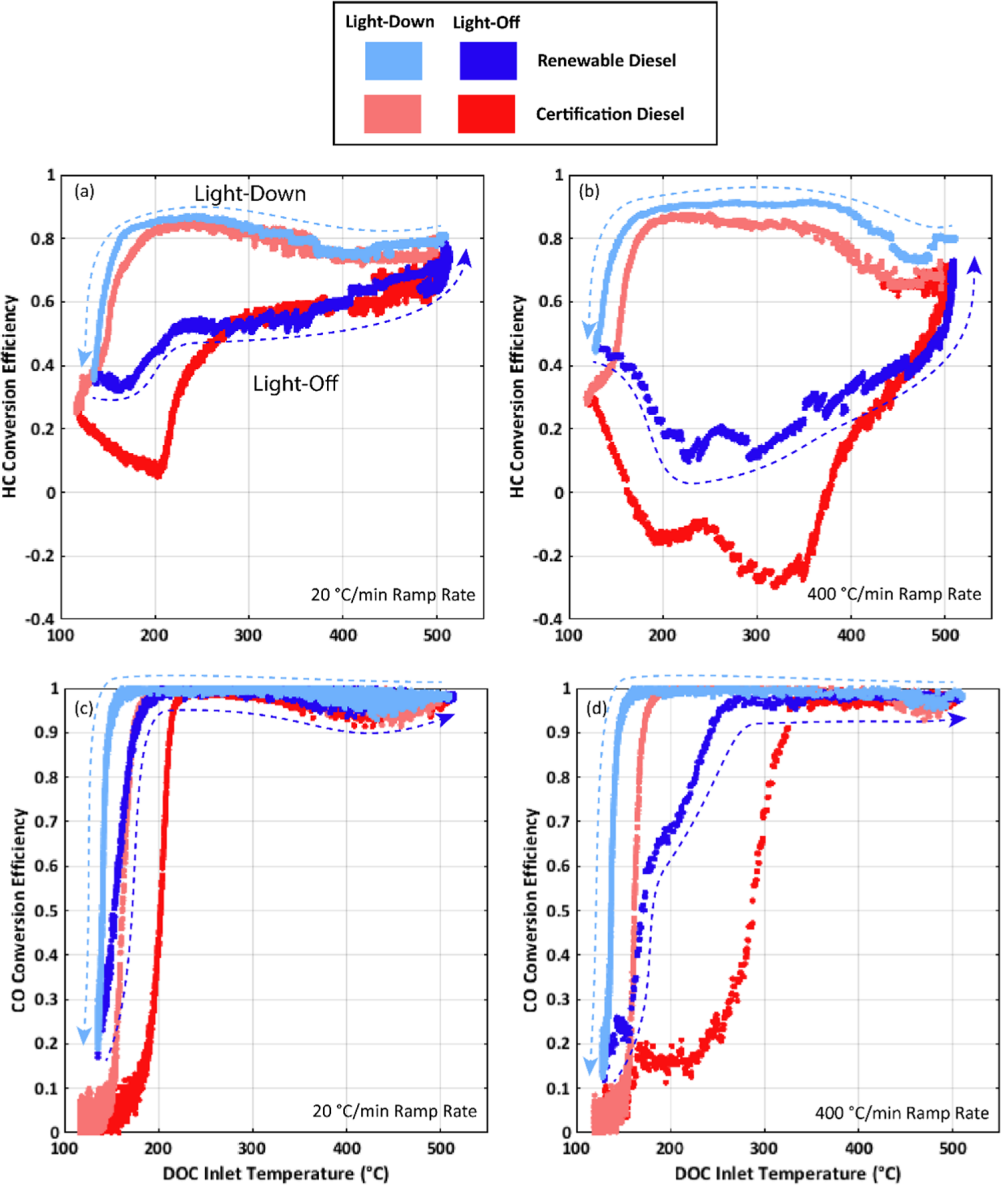
Comparison of conversion efficiencies during light-off and light-down as a function of the inlet DOC temperature for both fuels: (a) HC conversion efficiency during the 20 °C/min ramp, (b) HC conversion efficiency during the 400 °C/min ramp, (c) CO conversion efficiency during the 20 °C/min ramp, and (d) CO conversion efficiency during the 400 °C/min ramp.

Evaluation of HC and CO conversion during both the 20 °C/min and the 400 °C/min ramp rate is provided for the HVO renewable diesel and the 2007 certification diesel in Figure 6. During both ramp rates, the CO and HC conversion is shifted favorably for the HVO renewable diesel with respect to the 2007 certification diesel.

The benefit to DOC performance from using the HVO renewable diesel may come from multiple factors. As was noted in Figure 3, HC and CO engine-out emissions are higher for most of the ramp cycle for the 2007 certification diesel with respect to the HVO renewable diesel. Though HC and CO oxidation is complex, it is expected that increases in HC and CO emissions should reduce catalyst performance as competitive adsorption for limited active catalyst sites occurs. Various research works have reported reduced HC and CO conversion in the increased presence of one or both constituents.^14,40−42^

Additionally, the fuel chemistry differences between the two fuels should result in important differences in the unburned hydrocarbon composition in the exhaust as it relates to DOC performance. The differences in the distillation curves as provided in the supplemental information and highlighted in Table 2 suggest possible differences in the carbon chain lengths of the paraffinic portion of the fuel. Hydrocarbon chain length is another factor influencing DOC reactivity, with past research suggesting that the reactivity of n-alkanes tends to increase with the carbon chain length.^17,18,43^ Also, the HVO renewable diesel is devoid of aromatics, while the 2007 certification diesel has a 31.3% aromatic content. Past research has shown aromatics to negatively impact the conversion of other hydrocarbon species as aromatics have a high affinity for active surface sites and can block other HC species and oxygen, thereby limiting catalyst reactivity.^22^

It is difficult to determine in this work the exact cause of the improved DOC performance for the HVO renewable diesel with respect to the 2007 certification diesel, but it is likely a result of all three factors proposed: aromatic content, paraffinic hydrocarbon chain length, and engine-out levels of HC and CO.

It is also noted that for all fuels and all ramp rates, HC conversion efficiency during light-off first decreases unexpectedly as the inlet DOC inlet temperature increases and then later begins to increase as expected. Under certain conditions, such as the 2007 certification diesel and the 400 °C/min ramp rate, the HC conversion efficiency even drops to a negative number before increasing to its expected value. This may be explained by low-temperature HC storage and subsequent release at higher temperatures.^25,26^

**Figure 7:**
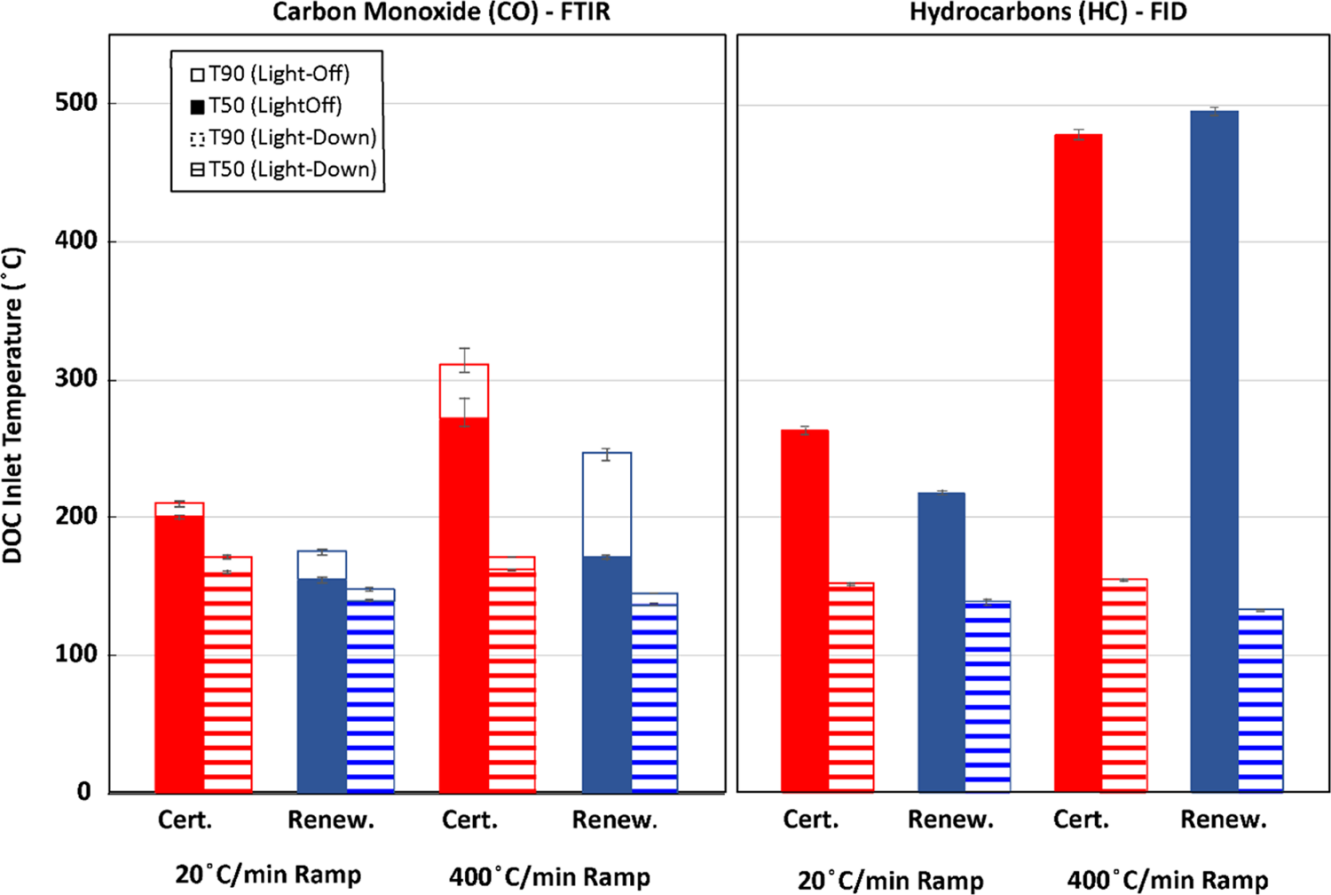
CO and HC light-off and light-down temperatures for both ramp rates and both fuels.

A summary of light-off and light-down performance with respect to T50 and T90 is given in Figure 7. T50 is noted as the temperature at which 50% conversion of the relevant constituent is reached and T90 is noted as the temperature at which 90% conversion of the relevant constituent is reached.

The HVO renewable diesel reduced T90 and T50 temperatures for nearly all conditions when considering both HC and CO. There was a single exception for HC emissions at the fastest ramp rate during the light-off condition. However, when reviewing the efficiency curves in Figure 6, it is clear that DOC performance throughout most of the ramp was significantly improved by the HVO renewable diesel, but that 50% conversion was reached at a similar temperature.

### Concluding Statements

## Supporting Information Available

Fuel distillation curves (ASTM D86) (distillation curves for both fuels) (Figure S1); fuel properties (listing of relevant fuel properties for both fuels) (Table S1); diagram of instrumented DOC (Figure S2); example of the temperature ramp profile for the 20 °C/min (a) and 400 °C/min (b) case (Figure S3); selected engine-out FTIR species comparison and total hydrocarbon comparison between FTIR and FID during the 20 °C/min ramp (Figure S4) (PDF)

## Abbreviations

BMEP: Brake mean effective pressure
CAI: California analytical instruments
CO: carbon monoxide
DOC: diesel oxidation catalyst
FAME: fatty acid methyl ester
FID: flame ionization detector
FTIR: Fourier transform infrared spectroscopy
HC: hydrocarbons
HVO: hydrotreated vegetable oil
LHV: lower heating value
MD/HD: medium- and heavy-duty
NDIR: nondispersive infrared
NO*_x_*: nitrogen oxides
T50: temperature of 50% conversion or 50% distillation
