# Supplementary material for: Impact of a Commercially Available Low Carbon Renewable Diesel Fuel on the Light-Off and Light-Down Characteristics of a Diesel Oxidation Catalyst
Source: ACS Omega. 2022 Aug 23;7(35):31367–76. doi: 10.1021/acsomega.2c03744 (PMC9453784; doi:10.1021/acsomega.2c03744)
Supplement: Supplementary file 1 — ao2c03744_si_001.pdf [file ao2c03744_si_001.pdf]

Impact of a Commercially Available Low Carbon Renewable Diesel Fuel on the Light-Off and Light-Down Characteristics of a Diesel Oxidation Catalyst

Jordan E. Easter, Martin L. Wissink, and Vicente Boronat Colomer*

Energy Science and Technology Directorate, Oak Ridge National Laboratory, P.O. Box 2008, Oak Ridge, TN 37831

Number of Pages: 4
Number of Figures: 3
Number of Tables: 1

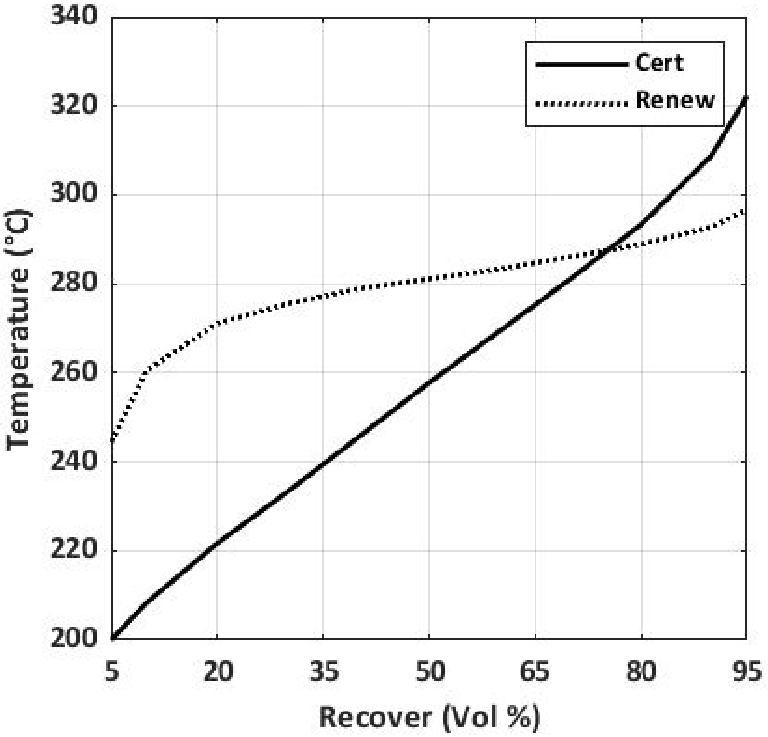

Figure S1. Fuel distillation curves (ASTM D86).

	Fuel	
	**2007 Cert. Diesel**	**HVO Renew. Diesel**
**Flash Point (℃)**	70.0	78.9
**Cloud Point (℃)**	-27.2	-26.0
**Viscosity, 40 ℃ (cSt)**	2.4	3.0
**Sulfur (ppm)**	9.1	< 0.5
**Carbon (wt%)**	86.7	84.6
**Hydrogen (wt%)**	12.9	15.0
**Naphthene (wt%)**	39.3	3.0

Table S1. Fuel properties.

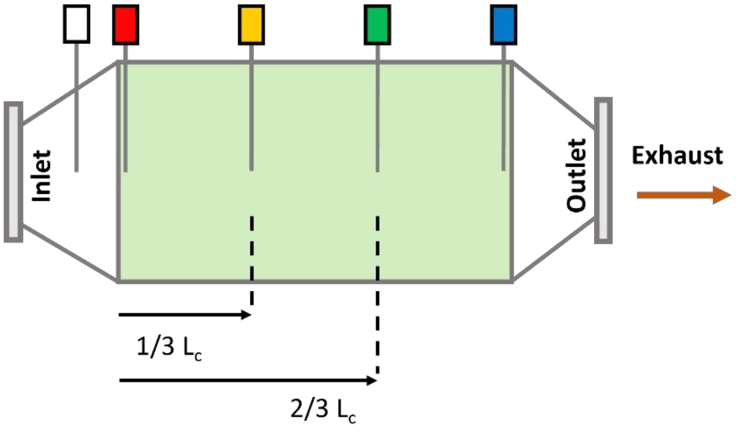

Figure S2. Graphic of instrumented DOC.

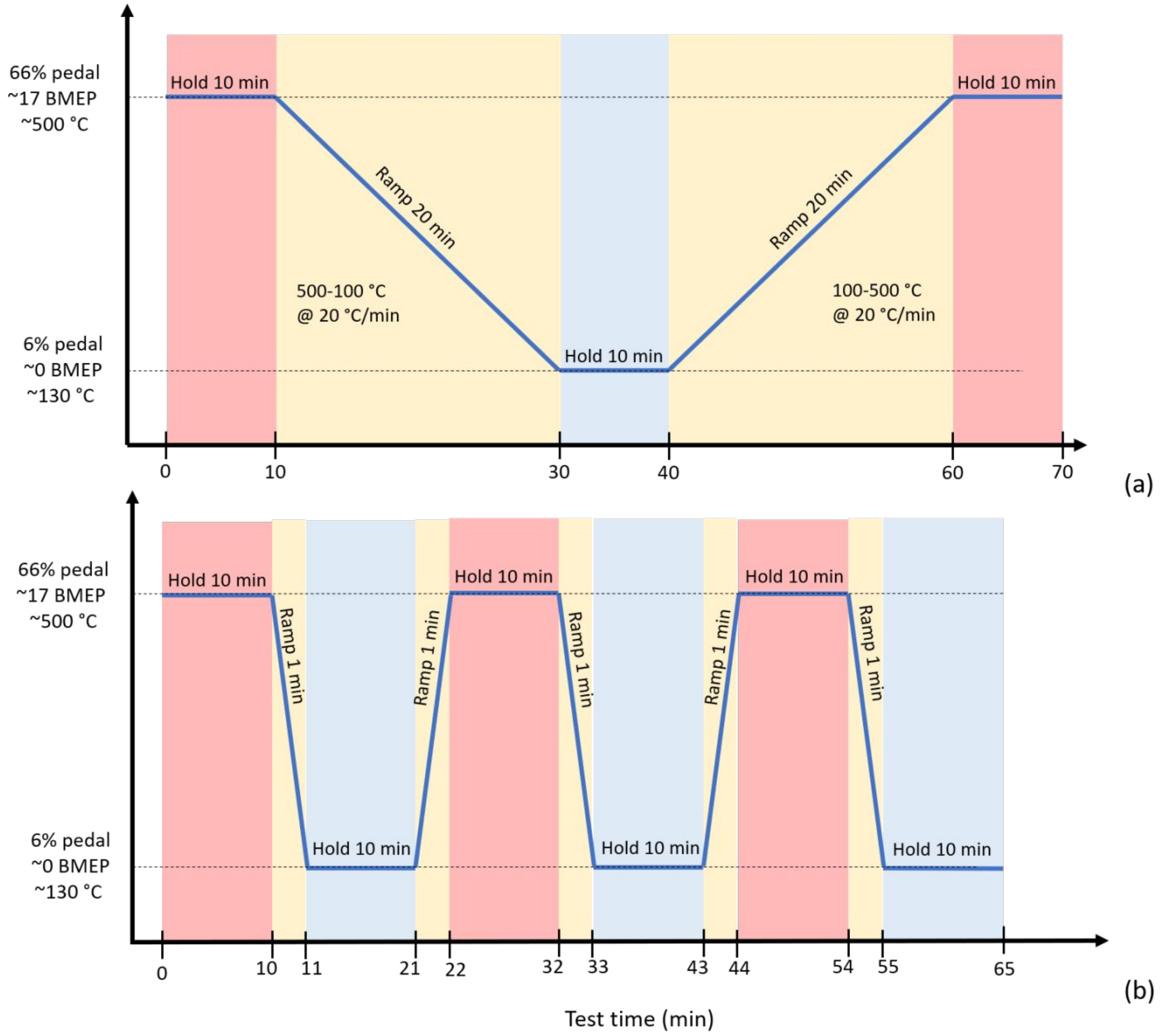

Figure S3. Example temperature ramp profile for the 20 °C/min (a) and 400 °C/min (b) case.

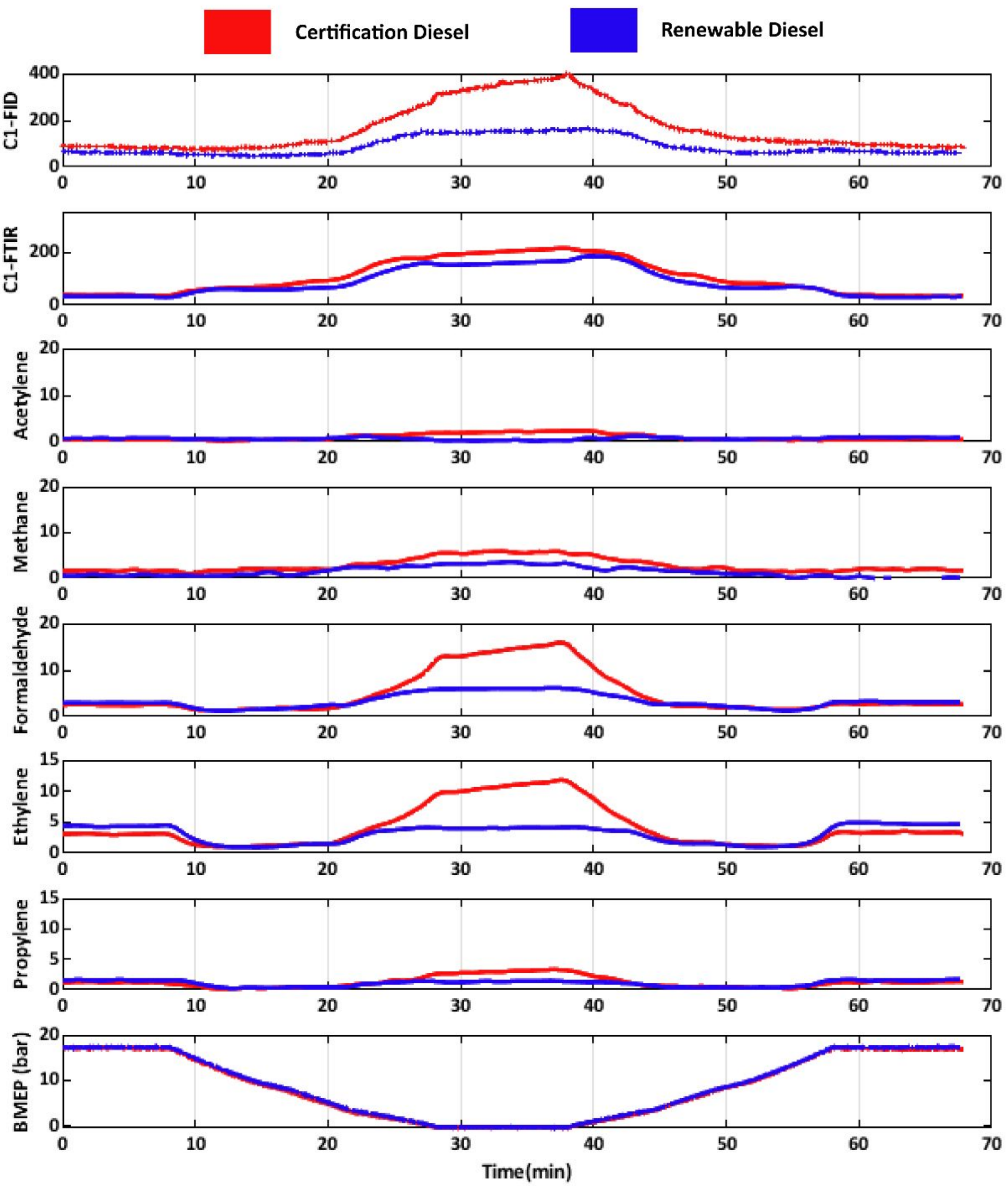

Figure S4. Selected engine-out FTIR species comparison and total hydrocarbon comparison between FTIR and FID during the 20 °C/min ramp.
